# Low-cost IoT real-time environmental monitoring and one-hour LSTM forecasting for small-scale agricultural applications

**DOI:** 10.1038/s41598-026-69489-0

**Published:** 2026-09-15

**Authors:** Ganna H. Hebishy, Ahmed K. Hassan, Samaa F. Osman, Mohamed S. Saraya

**Affiliations:** 1 Mechatronics Engineering Department, Faculty of Engineering, Mansoura University, Mansoura, 35516 Egypt; 2 Assistant Professor of Computers Engineering and Control Systems, Faculty of Engineering, Mansoura National University, Gamasa, 7731168 Egypt; 3 Electrical Engineering Department, Faculty of Engineering, Mansoura University, El-Mansoura, 35516 Egypt; 4 Computers Engineering and Control Systems Department, Faculty of Engineering, Mansoura University, Mansoura, 35516 Egypt

**Keywords:** Internet of things, Smart agriculture, Environmental monitoring, Long short-term memory, Time-series forecasting, Precision agriculture, Climate sciences, Engineering, Environmental sciences, Mathematics and computing

## Abstract

Standard equipment is too expensive and complex, frequently limiting continuous environmental monitoring in small-scale farming. To overcome this, a low-cost seven-channel IoT monitoring and forecasting system was developed and deployed, utilizing an Arduino Uno R3 and ESP8266 NodeMCU Wi-Fi module. The system acquired temperature, humidity, precipitation, soil moisture, methane, carbon monoxide, and air quality, transmitting data to the ThingSpeak cloud platform at an average interval of approximately one minute. The DHT22 temperature and humidity sensor carries a manufacturer-rated accuracy of ± 0.5 °C and ± 2–5% RH; the remaining sensors report uncalibrated raw output, as no reference-instrument validation was performed. The device was deployed for 21 days at a residential garden site in the Nile Delta region of Egypt. A total of 30,981 of 31,460 exported observations, 98.48% of the exported record were retained after the application of the preprocessing pipeline, which consisted of time-aware interpolation and Augmented Dickey-Fuller (ADF)-guided stationarity conditioning. A 70/15/15 chronological train/validation/test split was used to prevent temporal leakage. The analysis revealed distinct environmental patterns, including a rising trend of 0.296 °C/day, an anti-phase coupling between temperature and humidity, and an increase in raw gas sensor output overnight. To make use of this data for predictive insights, seven different univariate LSTM models were trained to forecast one hour ahead. When evaluated against persistence and seasonal-naive baselines on the same test set, the LSTM held a modest edge over persistence for methane (Mean Absolute Percentage Error, MAPE: 0.81%) and air quality (MAPE: 0.31%), while a simple persistence forecast matched or outperformed the LSTM for the remaining five channels. This field deployment demonstrates the technical feasibility of pairing affordable hardware with deep learning forecasting for continuous environmental monitoring, providing a foundation for future irrigation-support applications in resource-constrained settings.

## Introduction

Agriculture constitutes the foundation of human civilization, providing the food, economic security, and resource base essential for a growing population^1^. Agriculture has progressed through a series of changes driven by a single imperative: the need to produce more food with limited resources. The worldwide population is estimated to exceed ten billion by 2050, necessitating a minimum 50% increase in food production during this period to satisfy expected demand^1,2^. Simultaneously, cultivable land is diminishing due to urban expansion and soil deterioration, freshwater resources are becoming scarce amid competing needs and climatic pressures, and climate fluctuations are intensifying, collectively narrowing the window in which farmers must make increasingly critical decisions^2,3^. These converging pressures have driven the development of Agriculture 4.0, a data-centric model that integrates the Internet of Things, artificial intelligence, and cloud computing to transition agriculture from a reactive approach to a proactive, evidence-informed management strategy^2,4^.

The core concept of Agriculture 4.0 is the Internet of Things, including networked sensors, microcontrollers, and communication modules that enable ongoing, real-time collection of environmental data in agricultural environments^4–6^. IoT-enabled devices are capable of tracking temperature, humidity, soil moisture, precipitation, and atmospheric gas levels with high temporal precision, offering detailed environmental data that was previously unavailable to small-scale farmers^7,8^. The accessibility of economical microcontrollers like the Arduino Uno and low-power Wi-Fi modules such as the ESP8266 has increased the reach of this sensing framework beyond solely research institutions^9,10^, while cloud services like ThingSpeak offer data ingestion, storage, and real-time visualization without necessitating local server infrastructure^11,12^. Recent systematic evaluations confirm that affordable Arduino-class IoT stations have become a feasible instrument for ongoing environmental surveillance in resource-limited agricultural environments, with proven implementations across farms, greenhouse operations, and open-field locations^7,8^. Despite this advancement, high initial capital expenditures, connectivity constraints, and difficulties in data integration continue to limit widespread adoption, especially in small-scale and peri-urban agricultural settings^13^.

Despite the widespread use of IoT monitoring in agricultural studies, the majority of existing systems emphasize data collection rather than predictive functionality, leaving the rich temporal patterns present in sensor data underexploited^13^. Sensor data collected at elevated frequencies over prolonged durations exhibits significant autocorrelation, daily cycles, and longitudinal patterns, which might be utilized to forecast future environmental conditions and facilitate proactive rather than reactive agricultural management^14^. Long Short-Term Memory networks are highly suitable for this purpose: comprehensive evaluations of machine learning techniques for agricultural environmental time series consistently recognize LSTM as more effective than traditional statistical methods like ARIMA in modeling nonlinear temporal relationships and multi-scale dynamics^14^. The United Nations 2030 Sustainable Development Goals, especially SDG 2 (Zero Hunger), SDG 6 (Clean Water and Sanitation), and SDG 13 (Climate Action), highlight the critical need to transform raw sensor data into practical insights^15^, making the integration of machine learning into low-cost IoT pipelines a technically accessible and globally relevant path forward. Small-scale residential and peri-urban agricultural locations, especially in areas like the Nile Delta where climatic stress and resource limitations converge, are predominantly absent from documented implementations that pair field-deployed IoT data with trained predictive models^1,2,13^.

This study introduces an economical IoT system for environmental monitoring and forecasting, implemented during a 21-day period at a residential garden in the Nile Delta of Egypt. The system incorporates seven sensor channels that cover atmospheric, soil, and gas-phase measurements, transmits corresponding data to the ThingSpeak cloud platform roughly every minute, and utilizes univariate LSTM models trained on stationary time series to generate one-hour-ahead predictions for each channel separately. The contributions of this paper are as follows:

- A seven-channel detection system that incorporates atmospheric, soil, and gas sensors together with ThingSpeak cloud integration.
- A preprocessing pipeline attaining 98.48% data retention from 31,460 raw observations via time-sensitive interpolation and Augmented Dickey-Fuller (ADF) guided stationarity conditioning.
- Seven univariate LSTM models trained with ADF-guided differencing and evaluated at a fixed one-hour forecast horizon.
- Comprehensive performance evaluation employing Mean Absolute Error (MAE), Root Mean Square Error (RMSE), and Mean Absolute Percentage Error (MAPE) for all sensor channels, as well as bootstrap confidence intervals.

**Fig. 1:**
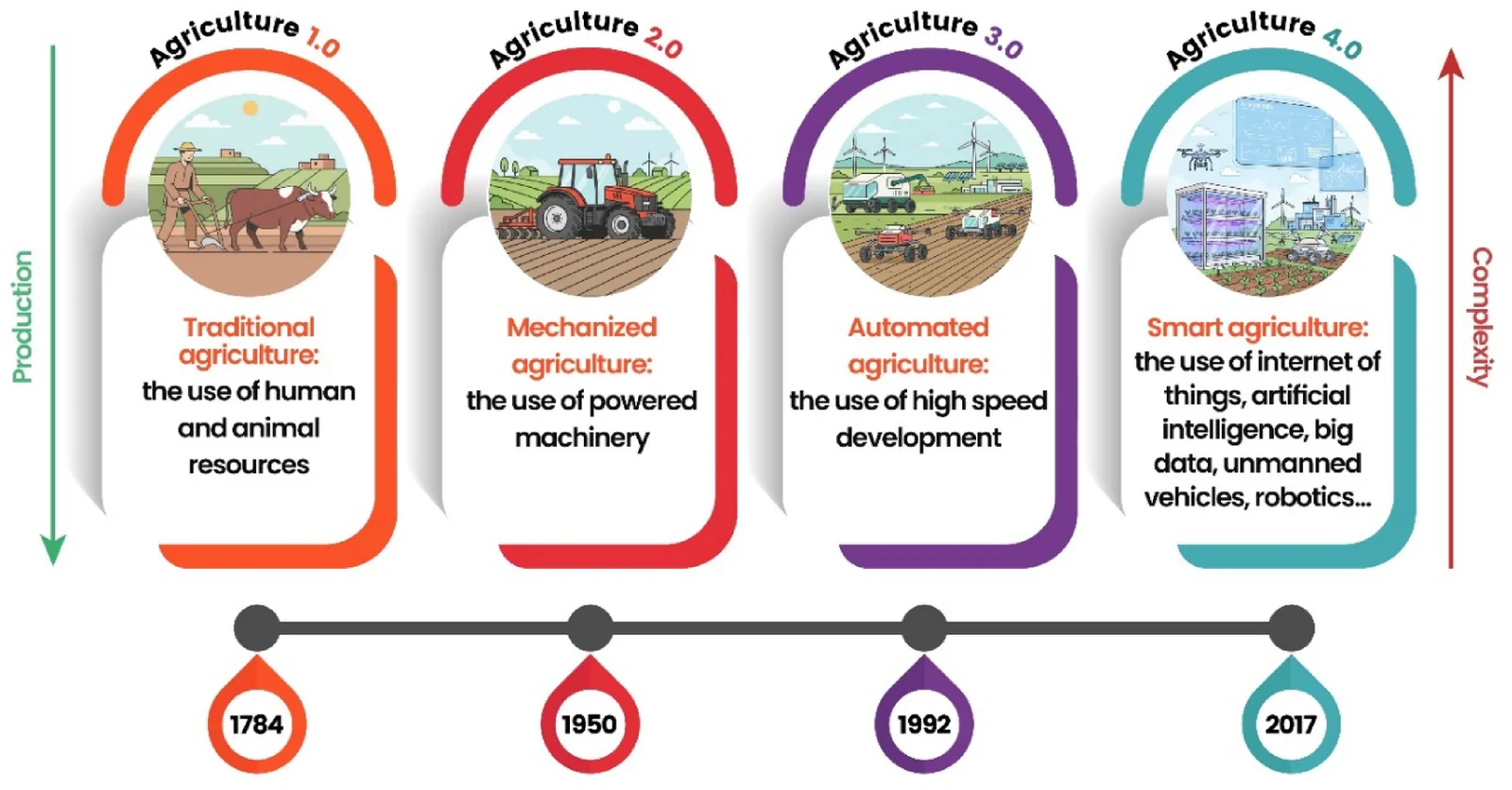
Progression of agricultural technologies from traditional to smart agriculture.

**Fig. 2:**
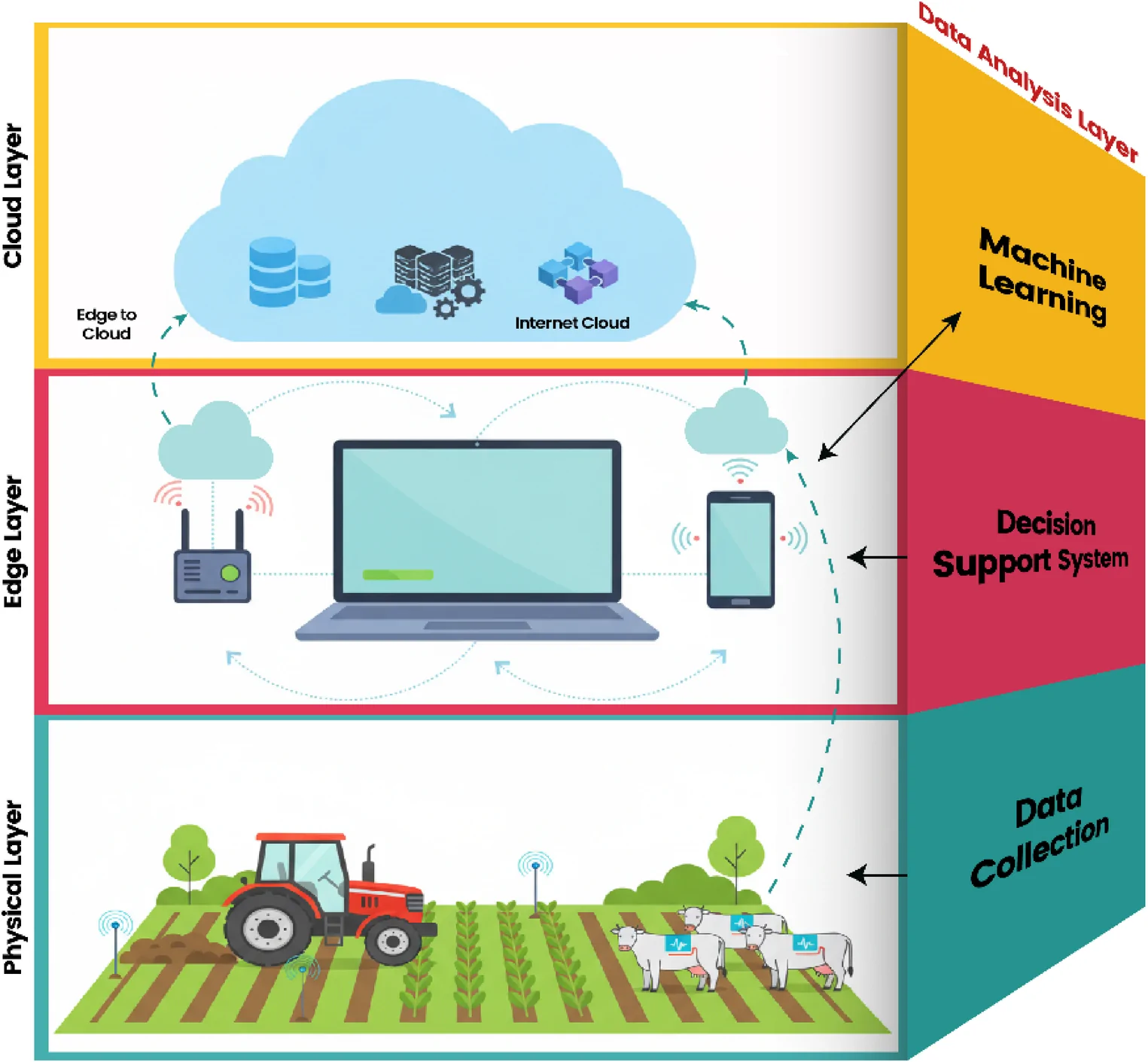
General three-layer IoT architecture for smart agricultural monitoring systems, illustrating the physical data collection layer, edge processing and connectivity layer, and cloud-based storage and machine learning analysis layer.

**Fig. 3:**
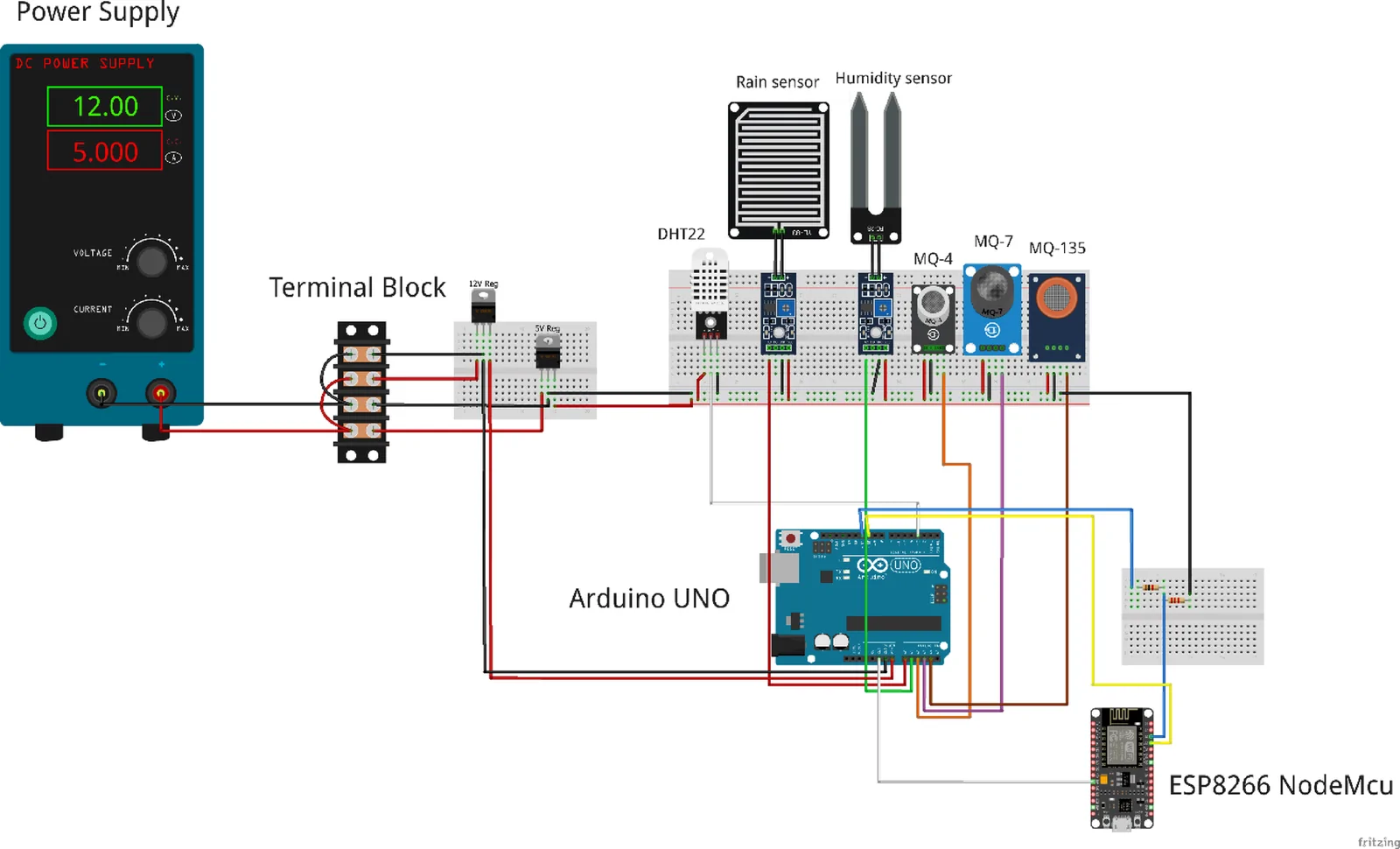
Circuit diagram of the proposed seven-channel IoT environmental monitoring system.

**Fig. 4:**
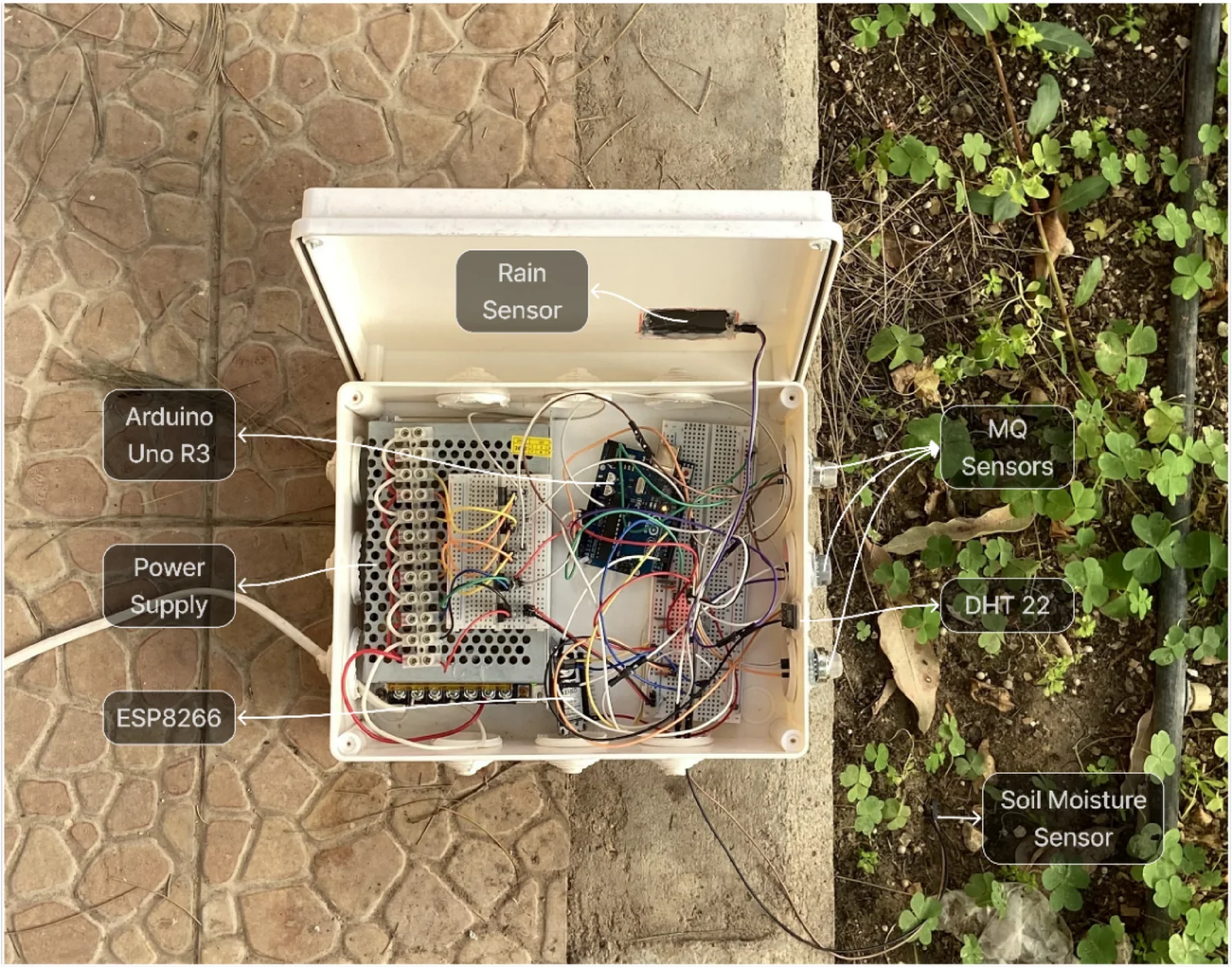
Top-down view of the internal hardware and breadboard assembly.

The remainder of this paper is organized as follows: Sect. 2 reviews related work; Sect. 3 describes the system architecture and methodology, including data preprocessing and the forecasting approach; Sect. 4 presents and discusses the results; and Sect. 5 concludes the paper (Figs. 1, 2, 3, 4).

## Related work

Low-cost IoT environmental monitoring and machine learning forecasting of environmental time series have advanced substantially in recent years, though these two lines of progress have largely run in parallel rather than converging in a single deployment. Hardware-focused work has demonstrated that Arduino-class and ESP-family platforms can capture multi-parameter field data and stream it to cloud platforms such as ThingSpeak, while a modeling line has established LSTM as consistently superior to classical statistical methods for environmental forecasting. The studies reviewed below are grouped by relevance to the present system, tracing how each combination of hardware, connectivity, and modeling approach has been applied across agricultural, industrial, and residential settings.

Mabrouki et al^9^. logged temperature, humidity, carbon monoxide, and sulfur dioxide from an Arduino Uno R3, ESP8266, DHT22, MQ-7, and MQ-136 to ThingSpeak, validating each sensor through laboratory experiments before a brief roadside trial. That Arduino Uno R3 and ESP8266 pairing with ThingSpeak, and the DHT22 and MQ-7 sensors, constitutes the closest hardware and cloud precedent to the present system, though the deployment was brief and offered only live monitoring. Narayana et al^10^. assembled an Arduino Uno station with GSM and Wi-Fi integrating sensors for temperature, humidity, pressure, rainfall, soil moisture, pH, turbidity, dissolved oxygen, conductivity, methane, ammonia, and hydrogen sulfide, publishing readings through an HTTP webpage for live visualization and alerting. The sensor breadth parallels this system closely, though no predictive model was applied to any channel.

Sazzad et al^16^. developed a low-cost capacitive IoT soil moisture sensor validated against oven-dry ground truth, using logarithmic and polynomial regression to predict water content and soil type with up to 96.49% accuracy. The low-cost sensor validation approach is consistent with the present system’s hardware philosophy, though the work was conducted under laboratory conditions rather than field deployment, and used regression rather than a trained sequence model.

Ramadan et al^17^. installed an IoT sensor node inside a chrome plating facility in Istanbul with detectors for ammonia, carbon monoxide, nitrogen dioxide, methane, carbon dioxide, sulfur dioxide, ozone, PM2.5, and PM10, evaluating LSTM, random forest, and linear regression per channel. The per-channel modeling and MQ-series gas sensing connect directly to the present design, though the industrial deployment excludes soil and precipitation channels. Banciu et al^18^. built a custom IoT device streaming temperature, humidity, PM2.5, and PM10 to ThingSpeak, training feedforward neural network and random forest models to forecast a composite air quality index, sharing the cloud backbone of the present system while collapsing all channels into a single target.

Elwakeel^8^ deployed a GSM-based automated monitoring and control unit inside poultry houses, tracking temperature, humidity, ammonia, and methane, with sensor readings validated against certified reference instruments (*r* > 0.96). The reference-instrument validation and gas-plus-climate sensing scope parallel the present system’s channel coverage, though the deployment targeted an enclosed livestock environment with automated control rather than open-field forecasting, and no LSTM or other predictive model was applied.

Filipovic et al.^19^ applied the Augmented Dickey-Fuller test before fitting ARIMA and LSTM to daily soil moisture records from a Serbian meteorological network, finding LSTM superior at short horizons; that ADF stationarity step is directly shared with the present work. Kumar and Rao^20^ compared SARIMA and LSTM for monthly soil moisture at three depths, finding LSTM superior across MAPE, MAE, and RMSE, a direct precedent for treating each environmental variable as its own univariate forecasting problem.

Alahmad et al.^21^ trained LSTM, random forest, and XGBoost models on IoT-collected soil moisture data across three soil types and five depths, finding LSTM competitive with tree-based ensembles for spatiotemporal soil moisture prediction. This is the closest methodological precedent for pairing IoT sensor data directly with LSTM forecasting, though the study modeled multiple depths and soil types jointly rather than treating each channel as an independent univariate series, and did not include gas or precipitation sensing.

Yang et al^22^. instrumented a solar greenhouse via ZigBee and GPRS with sensors for air temperature, humidity, soil temperature, soil moisture, light, and carbon dioxide, proposing a feed-forward attention LSTM at horizons of 12 to 48 h with jointly modeled inputs, in contrast to the one-hour-ahead univariate approach used here. Benyezza et al^23^. connected a Raspberry Pi node with an RF wireless sensor network monitoring temperature, humidity, soil moisture, and carbon dioxide through MQTT, applying fuzzy logic to automate irrigation and ventilation rather than a trained predictive layer.

Geng et al^24^. trained LSTM on ERA5-LAND reanalysis data to predict soil temperature, applying SHAP values and partial dependence plots for interpretability, without any physical sensing apparatus. Huang et al^25^. combined attention-LSTM with LightGBM for PM2.5 forecasting on four years of Beijing station data achieving R2 of 0.969, confirming LSTM’s strength on a gas-quality channel, though using archival data rather than field-deployed hardware. Kim et al^26^. validated the KOSEN weather station across 19 months of field operation in Japan, measuring temperature, humidity, rainfall, and wind via a WioLTE microcontroller with LTE, issuing threshold alerts without any forecasting component.

Across these studies, the hardware and modeling threads have advanced largely in parallel: the strongest LSTM designs tend to appear on archival datasets decoupled from field hardware, while the most field-validated low-cost IoT stacks are used for monitoring, alerting, or rule-based control. This system brings those threads together, pairing an Arduino Uno R3 and ESP8266 NodeMCU station transmitting seven sensor channels to ThingSpeak with ADF-validated univariate LSTM models forecasting one hour ahead for each channel independently over a 21-day outdoor residential deployment in the Nile Delta.

## System architecture and methodology

From data acquisition to forecasting, designing the proposed system involved two complementary considerations: a physical sensing infrastructure for collecting environmental data, and a computational pipeline for processing and modeling that data once acquired. The proposed environmental monitoring system is structured across three functional hardware layers: a physical sensing layer responsible for data acquisition, an edge processing layer that handles signal conditioning and transmission scheduling, and a cloud application layer that manages data ingestion, storage, and accessibility. This layered architecture reflects established design principles in IoT system engineering^1,27,28^, providing clear separation of concerns between hardware interfacing, local computation, and remote data management. Building on this hardware foundation, the methodology also covers how the collected data is cleaned and prepared, the statistical methods used to characterize its temporal behavior, and the design of the LSTM models used to forecast each sensor channel one hour ahead. The following subsections address each of these components in turn, beginning with the three hardware layers and concluding with the data processing and forecasting methodology.

### Data acquisition (the physical layer)

The physical sensing layer comprises an Arduino Uno R3 microcontroller interfaced with seven sensors mounted on a breadboard and housed within a 24 × 19 × 9 cm PVC dustproof, waterproof enclosure fitted with apertures to permit direct environmental exposure. The complete hardware bill of materials totaled 1,080 EGP (approximately $21 USD), underscoring the low-cost nature of the platform. The enclosure was placed at ground level in a shaded location, keeping all sensors within 10 cm of the ground and out of direct sunlight. The system was deployed in a residential garden in New Damietta City, Egypt, in the Nile Delta region, a semi-arid Mediterranean-influenced climate characterized by warm, dry summers and mild winters, providing a representative deployment context for small-scale agricultural monitoring applications. Power is supplied via a 12 V, 5 A adapter, ensuring continuous operation throughout the monitoring period. The estimated combined operating current draw of the sensing platform is approximately 610 mA (about 3 W) at 5 V, based on manufacturer-specified typical currents for each component and dominated by the three MQ-series sensor heaters (~ 150 mA each). No physical maintenance was performed during the deployment. The deployment included one planned downtime of approximately 57.7 h (5 to 7 May) to verify the connection was functioning correctly before data collection resumed. The remainder of the deployment also included periods with no successful transmission, examined further in Sect. 3.3.

The Arduino Uno R3 serves as the central controller, coordinating sensor polling across all seven channels and relaying encoded readings to the ESP8266 NodeMCU Wi-Fi module via a SoftwareSerial connection operating at 9600 baud. The complete hardware configuration is summarized in Table 1. Atmospheric temperature and relative humidity are acquired by a DHT22 sensor, which provides combined measurements within a single digital output with a stated accuracy of +/−0.5 °C and +/−2−5% RH. Surface precipitation is detected by the connected resistive rain sensor, and substrate moisture content is measured by a resistive soil moisture sensor. Ambient gas-phase concentrations are monitored by three MQ-series metal-oxide-semiconductor (MOS) gas sensors: the MQ-4 for methane (CH4), the MQ-7 for carbon monoxide (CO), and the MQ-135, its raw response sensitive to CO2, ammonia, benzene, and smoke. All MQ-series sensors require a resistive heating period following power-up before the sensing element reaches its rated operating temperature; a 30-second blocking delay is enforced in the Arduino firmware prior to the first sensor poll to ensure all MQ readings are captured within the sensor’s specified operating range. Each sensor was functionally tested prior to system assembly by exposing it to a stimulus and confirming a clear response followed by recovery to baseline: localized heat and humidity exposure for the DHT22, water contact for the rain and soil moisture sensors, and exposure to a combustible gas source for the MQ-series. This verified correct operation but did not constitute calibration against a reference standard or instrument, which was not performed for any sensor in this study; all reported values throughout the manuscript are therefore raw, uncalibrated sensor output.

**Table 1:** Comparative summary of reviewed studies.

Ref	Authors	Year	Hardware platform	Cloud/comms	Sensor types	Forecasting method	Key contribution	Gap relative to this work
^16^	Sazzad et al.	2025	Low-cost capacitive IoT soil moisture sensor	None	Soil moisture (capacitive), soil type	Logarithmic and polynomial regression	Low-cost sensor validated against oven-dry ground truth, 96.49% soil type prediction accuracy	Laboratory conditions only, no field deployment; regression rather than sequence model; single channel
^21^	Alahmad et al.	2025	IoT soil moisture sensors (multi-depth)	Not specified	Soil moisture (three soil types, five depths)	LSTM, random forest, XGBoost	LSTM competitive with tree-based ensembles for spatiotemporal soil moisture prediction	Multivariate/multi-depth joint modeling rather than per-channel univariate; no gas or rain sensing
^8^	Elwakeel	2025	GSM-based IoT monitoring/control unit	GSM	Temp, RH, NH3, CH4	None (automated control, no predictive model)	Reference-instrument-validated gas and climate sensing with automated control	Enclosed livestock environment, not open-field; no LSTM or forecasting; no soil or rain channels
^17^	Ramadan et al.	2024	IoT sensor nodes (industrial)	Cloud platform	NH3, CO, NO2, CH4, CO2, SO2, O3, PM2.5, PM10, temp, RH	LSTM, random forest, linear regression per channel	Industrial IoT air quality monitoring with per-channel ML forecasting and automated actuation	Industrial single-domain setting; no soil moisture or rain channels; no ADF testing
^18^	Banciu et al.	2024	Custom IoT air quality device	ThingSpeak	Temp, RH, PM2.5, PM10	Feedforward NN, random forest (composite AQI)	AIoT pipeline with ThingSpeak and ML forecasting	Single composite target; no per-channel LSTM; no soil, gas hazard, or rain channels
^10^	Narayana et al.	2024	Arduino Uno + GSM + Wi-Fi	HTTP webpage	Temp, RH, pressure, rain, soil moisture, pH, turbidity, DO, conductivity, CH4, NH3, H2S	None (live monitoring and alerting)	Broad multi-sensor real-time monitoring; sensor breadth closest to present system	No predictive model; water-quality channels outside this study’s scope
^24^	Geng et al.	2024	None (ERA5-LAND reanalysis)	None	Soil temperature (multi-depth, reanalysis-derived)	LSTM + SHAP, permutation importance, PDP	Explainable LSTM for soil temperature prediction	No physical IoT hardware; interpretability focus; reanalysis data only
^20^	Kumar and Rao	2023	None (archival station data)	None	Soil moisture (surface, profile, root, monthly)	SARIMA, LSTM	LSTM consistently outperforms SARIMA for soil moisture across three depths	No IoT hardware; monthly archival data; single variable; no atmospheric, gas, or rain sensing
^22^	Yang et al.	2023	ZigBee + GPRS agri-IoT network	Custom agricultural cloud	Air temp, air RH, soil temp, soil moisture, light, CO2	FAM-LSTM (feed-forward attention, multivariate)	Multivariate attention-LSTM for multistep greenhouse temperature and humidity prediction	Multivariate and multi-step; greenhouse only; no gas hazard or rain channels; no ADF testing
^23^	Benyezza et al.	2023	Raspberry Pi + RF wireless sensor network	MQTT broker + cloud server + Node-RED	Temp, RH, soil moisture, CO2	None (fuzzy logic actuation)	MQTT-connected greenhouse IoT with fuzzy rule-based irrigation and ventilation control	No LSTM or trained model; higher-power platform; greenhouse only; no gas hazard or rain channels
^25^	Huang et al.	2022	None (archival urban station data)	None	PM2.5, PM10, SO2, NO2, CO, O3, temp, pressure	LightGBM + LSTM-Attention ensemble	R2 = 0.969 for PM2.5; best-in-class ensemble attention-LSTM	No IoT deployment; archival data only; no soil or rain channels; ensemble rather than per-channel univariate
^26^	Kim et al.	2022	WioLTE microcontroller + LTE	Cloud server + LINE alerts	Temp, RH, rainfall, wind speed, wind direction, solar radiation	None (threshold alerting)	19-month continuous field deployment validating KOSEN weather station reliability	No forecasting model; no soil, gas, or particulate channels
^19^	Filipovic et al.	2021	24 national meteorological stations	National meteorological network	Daily temp, precipitation, vapour pressure deficit	ARIMA, LSTM (with ADF pre-testing)	ADF-validated LSTM outperforming ARIMA for regional soil moisture prediction	Regional network scale; no IoT hardware; no gas, particulate, or rain sensor channels
^9^	Mabrouki et al.	2021	Arduino Uno R3 + ESP8266 + Wi-Fi	ThingSpeak	DHT22 (temp, RH), MQ-7 (CO), MQ-136 (SO2)	None (live visualization and alerting)	Closest hardware and cloud precedent: Arduino Uno R3 + ESP8266 + ThingSpeak	Brief field trial only; no sustained deployment or forecasting component

**Fig. 5:**
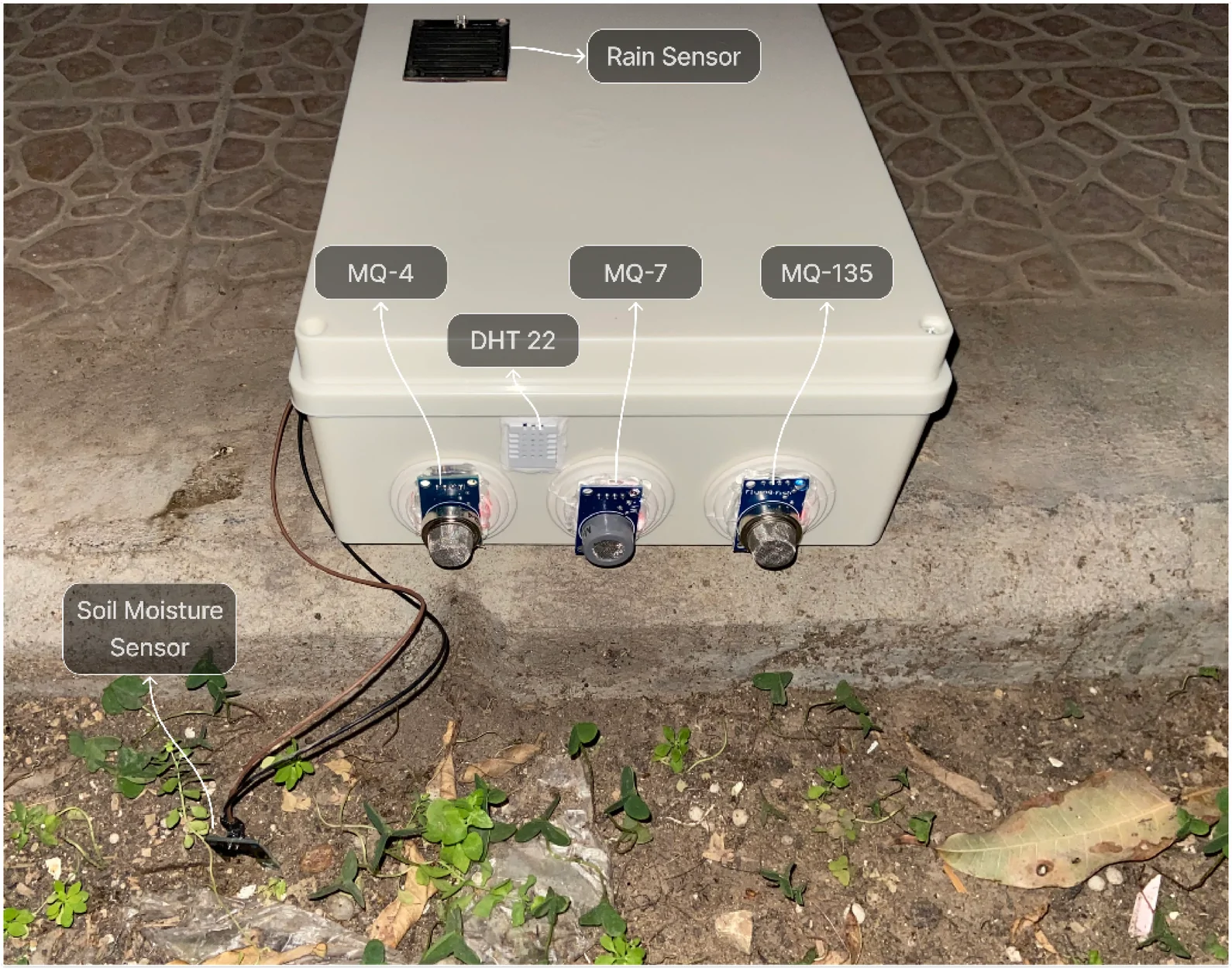
Profile view of the deployed sensing unit and external probes (MQ and soil moisture sensor).

### Signal processing (the edge layer)

Signal processing in this system is performed at the edge, on the Arduino Uno R3 and the ESP8266 NodeMCU, prior to cloud transmission, encompassing analog-to-digital conversion, targeted signal transformation, data serialization, and upload rate governance.

Raw analog readings from the rain and soil moisture sensors are inverted prior to serialization using the Arduino map() function, transforming the native ADC output (where 0 represents full saturation and 1023 represents dry conditions, owing to the inverse conductance characteristic of resistive sensors) to an intuitive scale in which 0 represents the dry or absent state and 1023 represents full wetness or saturation. This transformation was applied specifically to ensure that ThingSpeak dashboard visualizations remain interpretable to non-specialist stakeholders without domain knowledge of sensor electronics. All other sensor channels, including the DHT22 digital output and the three MQ-series analog outputs, are transmitted as acquired without additional scaling or filtering.

Sensor readings are serialized in CSV format in the fixed field order: humidity, temperature, rain, soil moisture, MQ-4, MQ-7, and MQ-135, and transmitted from the Arduino to the ESP8266 NodeMCU over the SoftwareSerial interface. A blocking delay in the Arduino main loop enforces the polling interval, acting as the primary rate-limiting mechanism. The ESP8266 NodeMCU firmware implements a secondary guard interval (MIN_INTERVAL), which prevents transmission in the event that a serial payload arrives before the preceding upload interval has elapsed. This dual-layer rate control ensures compliance with the ThingSpeak upload interval of 15 s while providing a conservative safety margin against rate-limit violations. Upon receiving a valid seven-field payload, the ESP8266 NodeMCU parses the data, validates the field count, and proceeds to the cloud transmission routine described in Sect. 3.3.

### Cloud integration (the application layer)

Cloud integration is implemented through the ThingSpeak IoT platform (MathWorks), operating under the free-tier service plan. ThingSpeak provides a RESTful HTTP API for time-series data ingestion, persistent storage, and retrieval, and was selected for its low configuration overhead, native multi-field channel support, and suitability for small-scale research deployments.

The ESP8266 NodeMCU transmits sensor data to ThingSpeak via HTTP POST requests directed to the platform write API endpoint (api.thingspeak.com, port 80), formatted as URL-encoded payloads containing the channel write API key and all seven field values. The connection is established using a native TCP socket via the Arduino WiFiClient library. Every successful write operation yields a numeric entry identifier from the ThingSpeak server; a response value higher than zero serves as the exclusive confirmation metric for a successful upload, after which the ESP8266 NodeMCU updates its internal timestamp and closes the connection. In the event of Wi-Fi disconnection, the firmware attempts automatic reconnection before any upload is attempted, with a bounded retry limit to prevent indefinite blocking.

ThingSpeak records each upload as a timestamped entry in UTC, with the channel configured to store all seven sensor fields. The full dataset was subsequently exported via the ThingSpeak bulk data export facility in CSV format for offline analysis. The firmware’s polling routine targets a 20-second cycle, enforced by a blocking delay in the Arduino main loop, which would yield approximately 225 observations per hour and 4,320 per day under continuous, uninterrupted transmission. The achieved cadence was closer to one minute in practice, reflecting per-upload network overhead together with the planned downtime and periods with no successful transmission thereafter. Including these periods, the deployment produced 31,460 raw observations across the 21-day window, approximately 1,498 per day, one every 58 s on average, of which 30,981 were retained after preprocessing, 98.48% of the exported record. Measured against the 20-second design target rather than the achieved cadence, the deployment captured 34.68% of the theoretically possible 90,720 observations (34.15% after preprocessing); the 98.48% figure above reflects retention relative to the exported record, not schedule-based completeness.

### Data processing and forecasting

The raw data collected through the ThingSpeak cloud platform required a structured sequence of preprocessing, statistical characterization, and model development steps before forecasting could be conducted. This subsection describes each of these steps as implemented, covering data ingestion and cleaning, stationarity-conditioned temporal analysis, and LSTM model design and training.

#### Data preprocessing pipeline

Upon ingestion, timestamps were converted to UTC + 3 to align all temporal indices with local solar time, and ThingSpeak field identifiers (field1 through field7) were remapped to descriptive column names: humidity, temperature, rain, soil moisture, MQ-4, MQ-7, and MQ-135.

DHT22 fault readings were corrected by enforcing physical plausibility bounds of [0, 60] °C for temperature and [0, 100]% for humidity, with out-of-bounds values replaced using time-aware linear interpolation. MQ-series fields were screened and corrected for zero-valued warm-up artefacts using the same approach. A maximum consecutive gap limit of 20 observations (approximately 5.3 min) was applied; rows exceeding this limit were removed.

#### Statistical and temporal analysis methods

Prior to model development, a structured analysis was conducted across all seven sensor fields to characterize their statistical properties, longitudinal behavior, and diurnal dynamics. All analyses were applied to the cleaned dataset produced in Sect. 3.4.1.

Stationarity was assessed using the Augmented Dickey-Fuller (ADF) test applied independently to each of the seven fields. The ADF test evaluates the null hypothesis that a unit root is present in a time series; rejection of the null hypothesis at a significance level of *p*< 0.05 indicates a stationary series suitable for modeling in levels. Fields that failed to reject the null hypothesis were identified as non-stationary and flagged for first-order differencing prior to model input construction. Although LSTM networks do not require stationarity to the same extent as classical autoregressive models, differencing was applied to reduce the extent to which the network must extrapolate to absolute values outside its training range, following the same ADF-guided approach used by Filipovic et al^19^. for LSTM-based soil moisture forecasting.

Longitudinal trends were characterized using linear regression applied to hourly-resampled series for each field, with statistical significance evaluated at *p* < 0.001. Seasonal-trend decomposition using locally estimated scatterplot smoothing (STL) was additionally applied to extract the trend component independently of the seasonal and residual elements. Diurnal dynamics were characterized by computing mean sensor profiles aggregated by hour of day across the full observation window, using UTC + 3 local time.

#### LSTM model design and training

A Long Short-Term Memory network was selected as the forecasting architecture for all seven sensor fields. LSTM networks are well suited to environmental sensor time series due to their capacity to retain long-range temporal dependencies through gated memory cells, handling the autocorrelation structure, diurnal periodicity, and trend non-stationarity that are characteristic of continuously recorded environmental data. Univariate models were trained independently per field to avoid cross-field gradient interference given the heterogeneity of the sensor signals. A standard stacked LSTM was used rather than a modified variant such as Haq’s CDLSTM^29^, developed for long-range climate forecasting, or SMOTEDNN^30^, developed for air pollution classification, since the per-field univariate design already isolates each channel’s behavior without requiring additional architectural modification. A two-layer stacked architecture with 64 and 32 hidden units, a dropout rate of 0.2, and a fully connected output layer mapping to 60 forecast steps was applied uniformly across all fields. Training used the Adam optimizer with a learning rate of 0.001, mean squared error loss, and early stopping with a patience of 10 epochs monitored on validation loss. This architecture totals 31,676 trainable parameters per model, identical across all seven fields.

Raw observations, collected at an average interval of approximately one minute, were resampled to a uniform one-minute resolution using mean aggregation. The three non-stationary fields identified in Sect. 3.4.2 were first-order differenced before scaling to ensure stationarity during training; evaluation metrics for these fields were subsequently reconstructed in original physical units by anchoring each forecast window independently to the true raw observation at the end of that window’s own lookback period. All fields were independently normalized to [0, 1] using MinMax scaling fitted on the training portion only to prevent data leakage. Overlapping input-output sequences of 120 input steps and 60 output steps were constructed and assigned to a chronological 70/15/15 train/validation/test split. The test set was not accessed until final evaluation. Windows adjacent to a split boundary share up to 179 of 180 raw timesteps with their neighbor, though this affects only 5.4% of validation and test windows and does not measurably change baseline error when excluded.

Model performance was assessed on the held-out test set using three complementary metrics: Mean Absolute Error (MAE), Root Mean Square Error (RMSE), and Mean Absolute Percentage Error (MAPE). MAE quantifies the average magnitude of forecast errors in original physical units, RMSE penalizes larger errors more heavily due to the squared term, and MAPE expresses error as a percentage of the actual value, facilitating comparison across fields with different measurement scales.

## Results and discussion

### System deployment and data acquisition outcomes

**Fig. 6:**
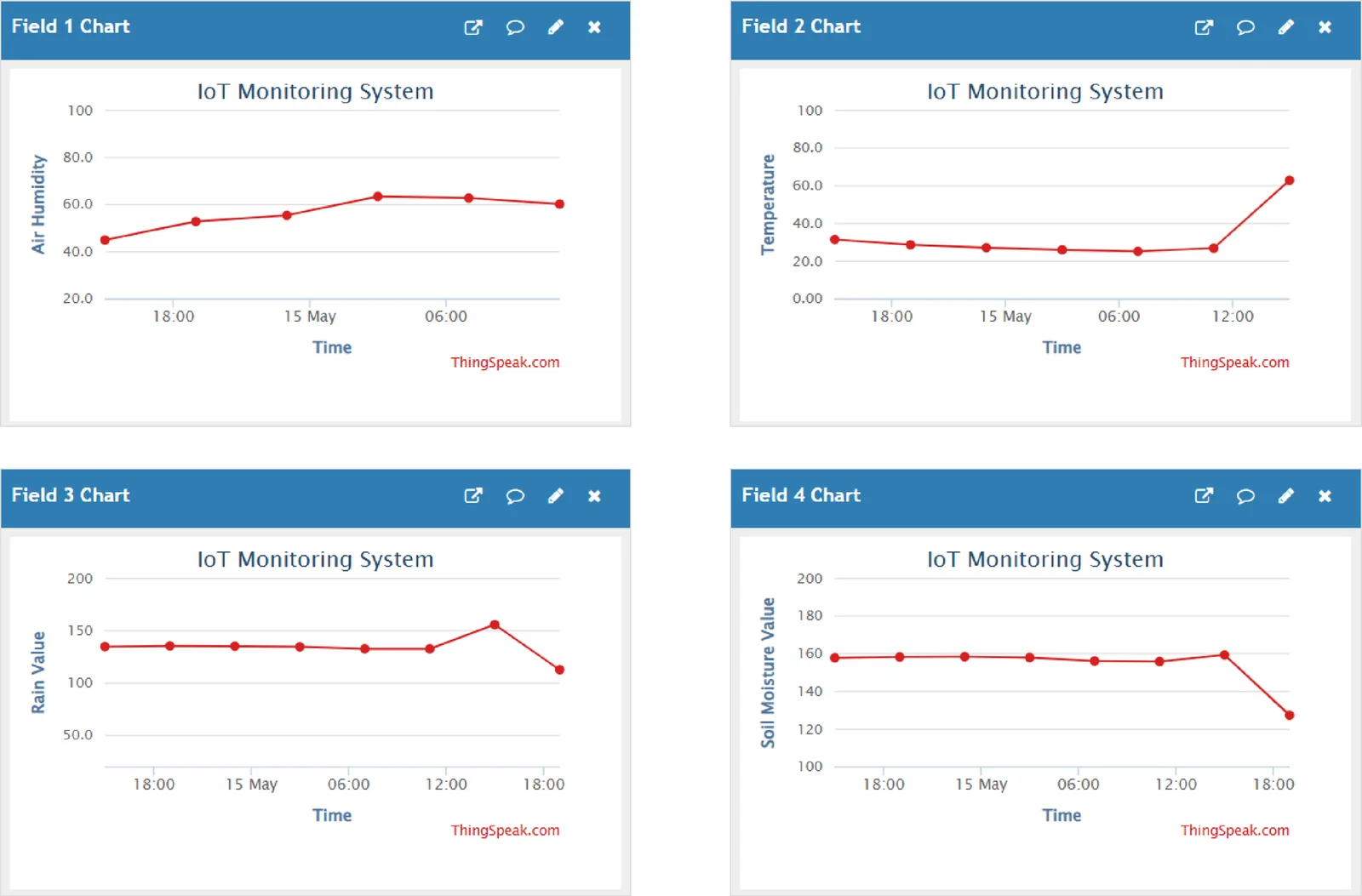
ThingSpeak cloud dashboard interface showing the humidity, temperature, rain value, and soil moisture value confirming live cloud transmission during deployment.

The system operated across the 21-day deployment window, spanning 24 April to 15 May 2026, transmitting all seven sensor channels to the ThingSpeak cloud platform at an average interval of approximately one minute. The ThingSpeak dashboard provided real-time visualization of all seven fields through automatically updated channel charts (Figs. 5 and 6). Connectivity was paused once for approximately 57.7 h to verify the connection was functioning correctly before data collection resumed. Temperature and humidity readings reflected consistent diurnal cycling, with daily peaks near 13:00 and humidity troughs near 12:00 local time. MQ-7 and MQ-135 displayed a morning elevation pattern consistent with nocturnal gas accumulation and convective dispersal, while rain and soil moisture channels showed corresponding raw-signal changes during periods of rainfall. The cloud-based architecture eliminated the need for local storage and enabled remote access without additional infrastructure, confirming the practical viability of the deployment approach for resource-constrained settings.

**Fig. 7:**
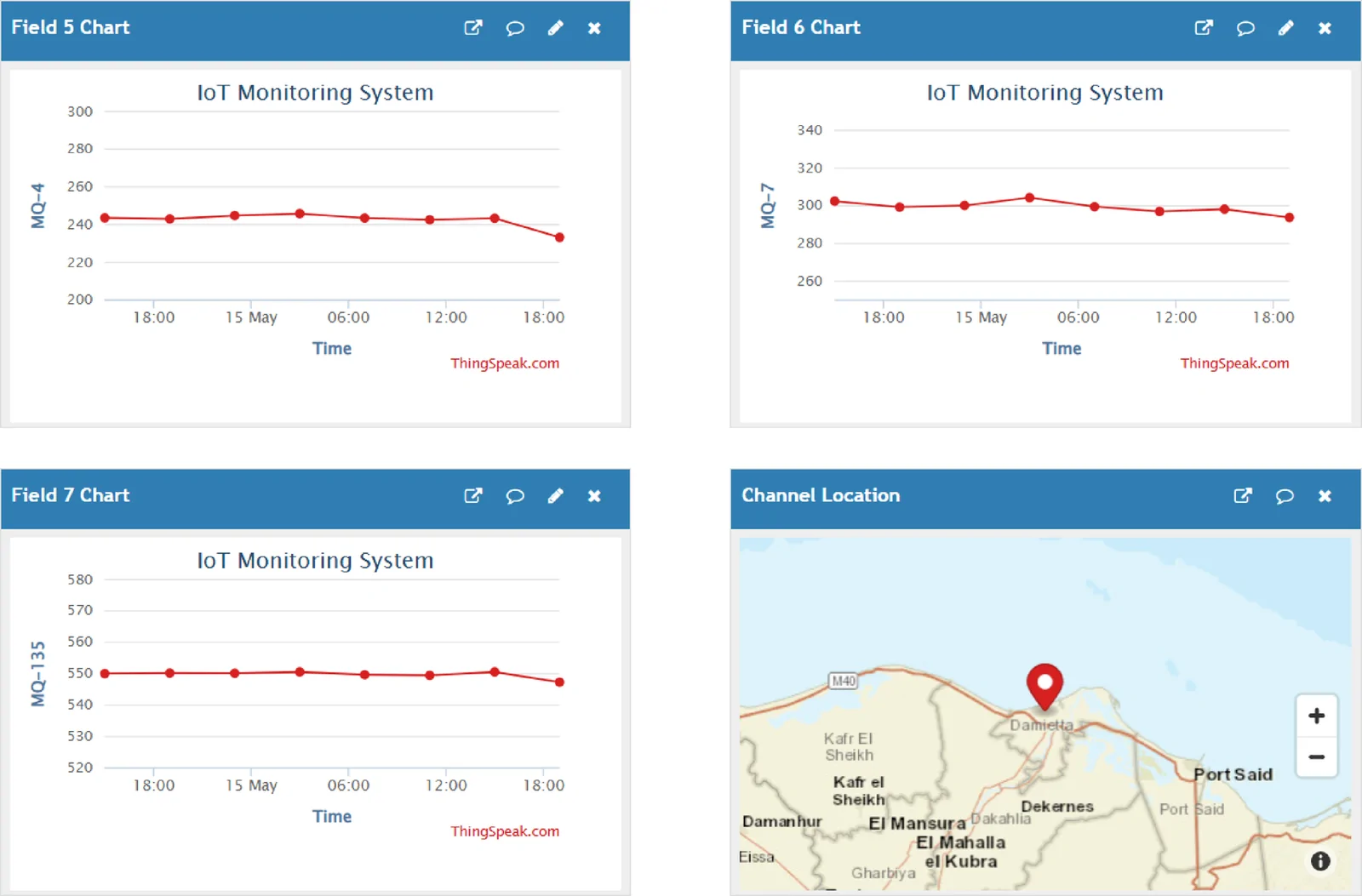
ThingSpeak cloud dashboard interface showing the air monitoring parameters (MQ-4, MQ-7, and MQ-135) along with the location confirming live cloud transmission during deployment.

The raw export contained 31,460 observations. DHT22 fault readings flagged 890 temperature observations (2.83%) and 682 humidity observations (2.17%) as out-of-bounds. Time-aware linear interpolation recovered 411 temperature and 364 humidity observations, while 479 rows exceeding the maximum consecutive gap limit were removed. The final cleaned dataset retained 30,981 observations, representing a retention rate of 98.48%. Post-cleaning, temperature spanned 3.0 to 57.1 °C (mean 23.17 °C, std 3.87 °C) and humidity spanned 7.3 to 90.9% (mean 66.14%, std 13.04%). The upper end reflects a brief warming episode in the final days of the deployment; the enclosure was shaded throughout. No zero-valued warm-up artefacts were detected in any MQ-series field. The complete preprocessing pipeline and its outcomes are summarized in Table 2.

**Table 2:** Hardware configuration: physical layer components.

Component	Type	Specifications	Function
Arduino Uno R3	Microcontroller	ATmega328P, 16 MHz, 14 digital I/O, 6 analog inputs	Main controller; sensor polling and serial data relay
ESP8266 NodeMCU	Wi-Fi Module	802.11 b/g/n, SoftwareSerial UART at 9600 baud	Wireless data transmission to ThingSpeak cloud platform
DHT22	Temp. and humidity	Range: −40 to + 80 °C, 0–100% RH; accuracy: +/−0.5 °C	Ambient temperature and relative humidity acquisition
Rain sensor	Precipitation	Analog resistive, A0; mapped 0 (dry) to 1023 (wet)	Surface precipitation detection
Soil moisture sensor	Soil moisture	Analog resistive, A1; mapped 0 (dry) to 1023 (saturated)	Substrate moisture content monitoring
MQ-4	Methane (CH4)	Analog output, A2; metal-oxide-semiconductor (MOS)	Methane gas concentration monitoring
MQ-7	Carbon monoxide (CO)	Analog output, A3; metal-oxide-semiconductor (MOS)	Carbon monoxide concentration monitoring
MQ-135	General gas sensor output	Analog output, A4; metal-oxide-semiconductor (MOS); sensitive to CO2, NH3, benzene, smoke	General gas sensor monitoring

### Environmental pattern and trend analysis

Stationarity was assessed independently for each of the seven sensor fields using the Augmented Dickey-Fuller test. Four fields, temperature, humidity, rain, and MQ-7, rejected the null hypothesis and were modeled in levels. The remaining three, soil moisture, MQ-4, and MQ-135, failed to reject the null hypothesis and were identified as non-stationary, requiring first-order differencing prior to model input construction. Full ADF statistics are reported in Table 3.

**Table 3:** Preprocessing pipeline stages and outcomes.

#	Stage	Operation	Outcome
1	Ingestion and timezone conversion	Parse timestamps; convert to UTC + 3; rename field identifiers	31,460 rows loaded; index aligned to local time
2	DHT22 plausibility bounds	Flag temperature outside [0, 60] °C and humidity outside [0, 100]%	890 temperature faults (2.83%); 682 humidity faults (2.17%)
3	MQ warm-up zero detection	Flag zero-valued readings in MQ-4, MQ-7, and MQ-135	No zero-value readings detected
4	Time-aware linear interpolation	Interpolate with elapsed-time weighting; max gap limit 20 observations (~ 5.3 min)	411 temperature and 364 humidity observations recovered
5	Unrecoverable row removal	Drop rows where temperature or humidity remain missing after interpolation	479 rows removed; 30,981 rows retained (98.48%)

**Fig. 8:**
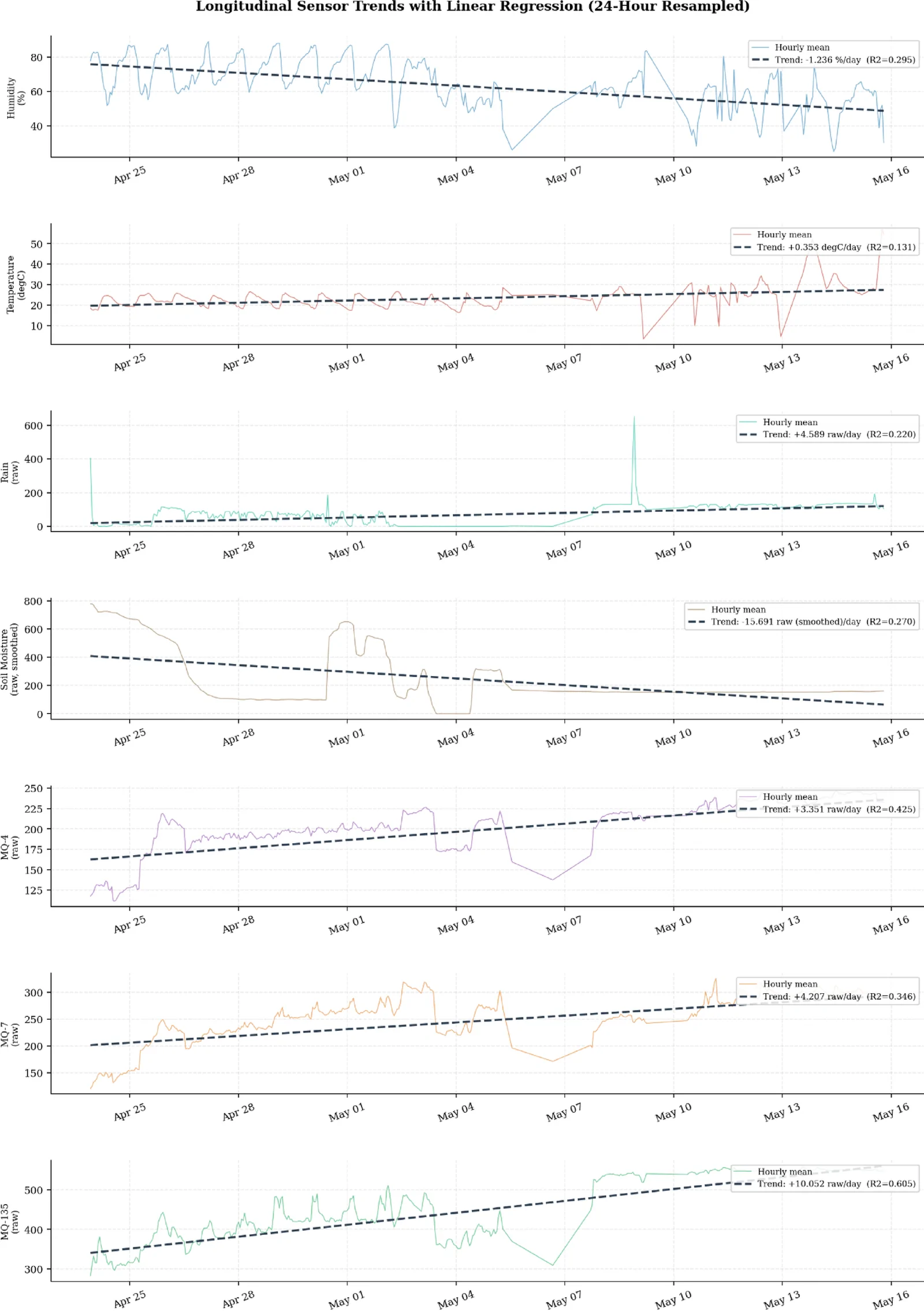
Longitudinal trends for all seven sensor fields plotted on hourly-resampled series with linear regression trend lines.

Linear regression applied to hourly-resampled series confirmed statistically significant longitudinal trends across all seven fields (*p* < 0.001). Temperature rose at + 0.353 °C per day, corroborated by the STL-extracted trend component at + 0.296 °C per day (R2 = 0.651), representing a net warming of 5.15 °C over the 21-day window. Humidity declined correspondingly at −1.236% per day, consistent with the inverse psychrometric relationship under rising ambient temperature. MQ-135 exhibited the strongest longitudinal signal across all fields at + 10.052 raw units per day (R2 = 0.605), reflecting a sustained upward trend in raw output throughout the monitoring period. MQ-7 and MQ-4 rose at + 4.207 and + 3.352 raw units per day respectively. Soil moisture readings exhibited sharp, short-lived spikes attributable to manual watering events rather than continuous environmental dynamics; a centered rolling median filter (450-observation window) was applied to suppress these spikes prior to trend and diurnal analysis, affecting 545 observations (1.8% of the record) while leaving the series median unchanged (156.0 raw units, both raw and smoothed). Longitudinal trends for all fields are visualized in Fig. 7, and the STL decomposition components for temperature are presented in Fig. 8.

**Fig. 9:**
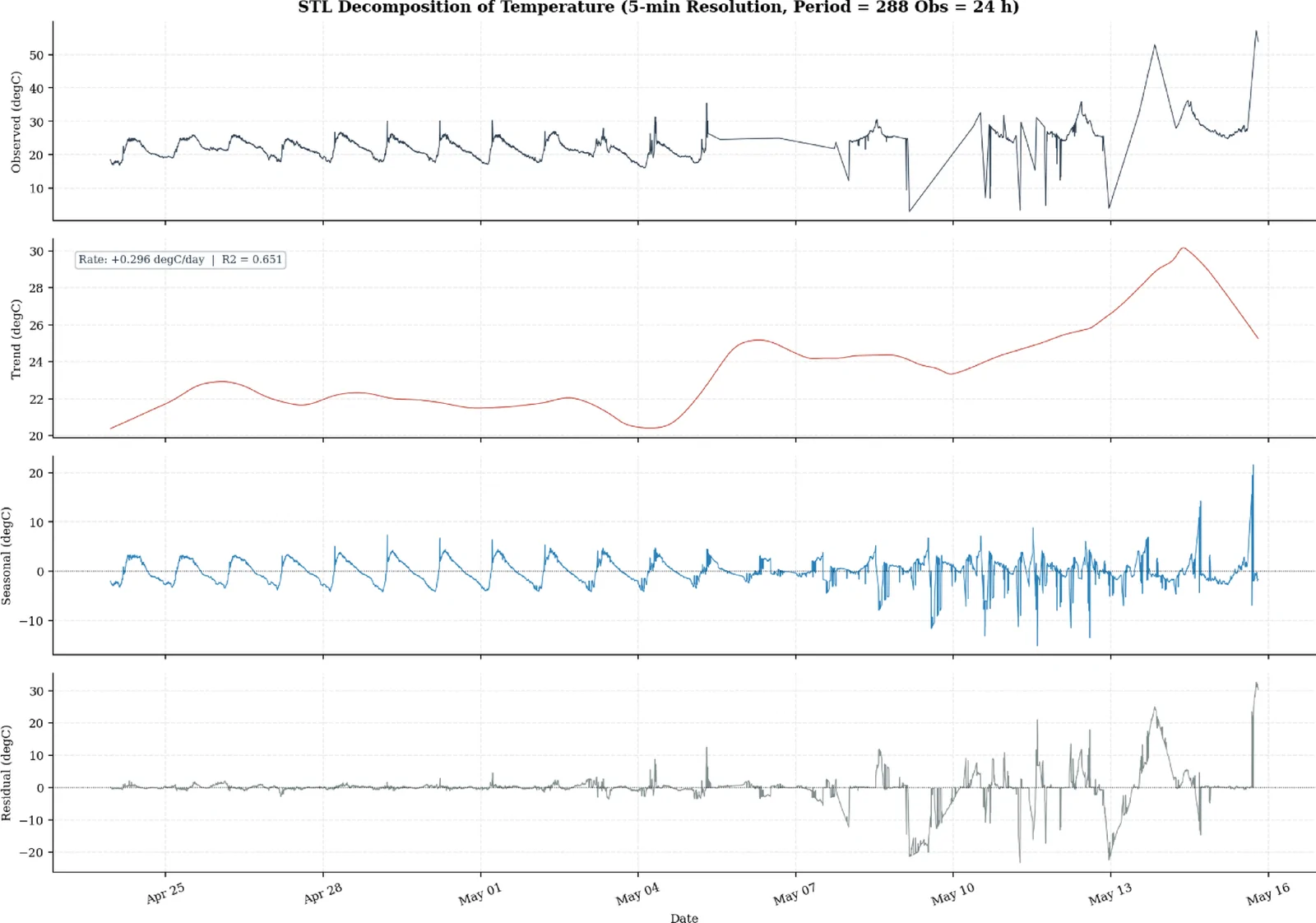
STL decomposition of the temperature series showing observed, trend, seasonal, and residual components.

Mean sensor profiles aggregated by hour of day across the full observation window revealed consistent diurnal dynamics across all seven fields (Fig. 9). Temperature peaked at 26.42 °C at 13:00 and reached a minimum of 20.04 °C at 04:00, yielding a daily range of 6.38 °C. Humidity followed a strict anti-phase pattern, peaking at 76.47% at 04:00 and falling to 55.15% at 12:00, a range of 21.32% points, with the humidity trough lagging the temperature peak by approximately one hour due to the thermal inertia of the near-surface air mass. MQ-7 and MQ-135 raw output both peaked at 07:00, recording mean values of 259.67 and 463.93 raw units respectively, before declining through the morning, a pattern that may plausibly reflect nocturnal near-surface gas accumulation. Both fields reached their daily minima at 02:00. MQ-4 exhibited a distinct nocturnal peak at 23:00 (205.45 raw units), diverging from the morning peak profile of the other gas sensors, a pattern that could plausibly reflect near-surface soil outgassing. Soil moisture peaked at 02:00 (324.30 raw units) and reached its minimum at 11:00 (219.13 raw units), with the morning trough occurring as temperature rose and humidity fell, broadly consistent with rising evapotranspiration demand. Rain sensor output was highest during nocturnal hours, peaking at 00:00 (78.22 raw units), consistent with predominantly night-time precipitation across the monitoring window.

**Fig. 10:**
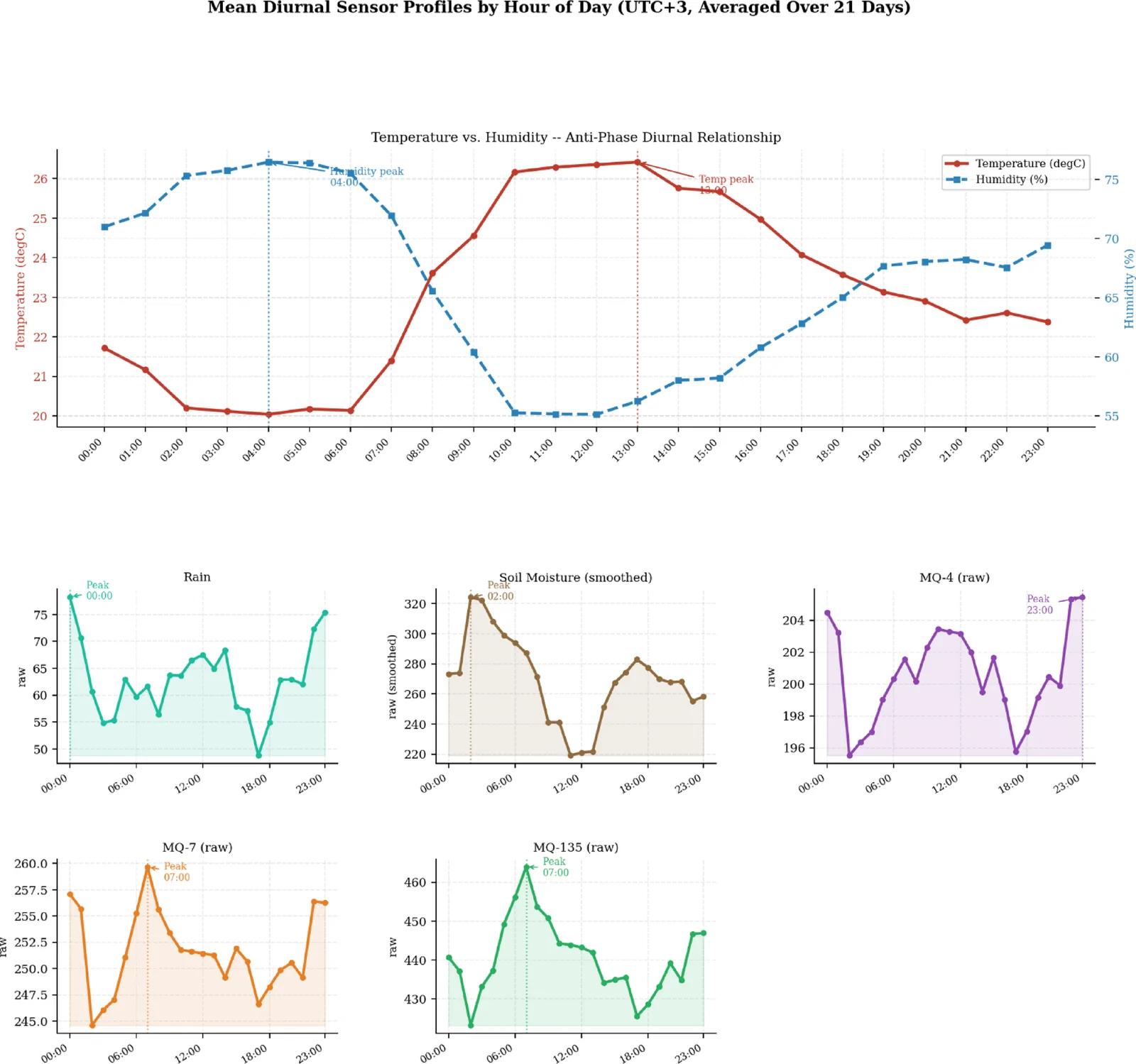
Mean diurnal profiles by hour of day (UTC + 3, 21-day average). Top: temperature and humidity on twin axes. Bottom: rain, soil moisture, MQ-4, MQ-7, and MQ-135.

**Fig. 11:**
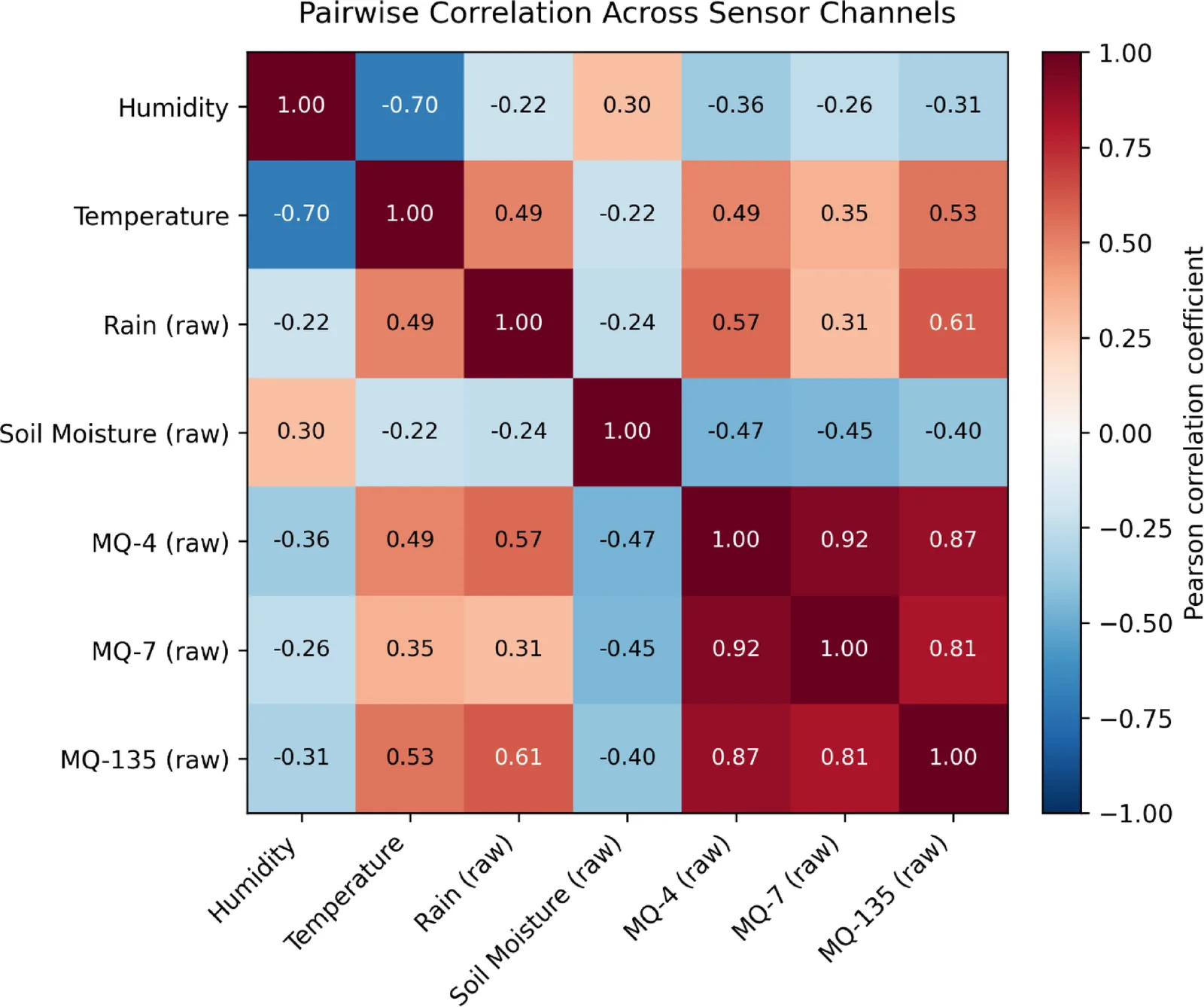
Pairwise Pearson correlation matrix across all seven sensor channels, computed over the full 21-day deployment record (*n* = 30,981 observations).

To characterize coupling across channels, Fig. 10 presents the pairwise Pearson correlation matrix computed across the full deployment record. The three MQ-series channels are strongly intercorrelated (*r* = 0.81 to 0.92) and each tracks positively with temperature and rain and negatively with humidity and soil moisture, consistent with a shared response to ambient conditions. The strongest single pairwise relationship is the anti-phase coupling between temperature and humidity (*r* = −0.70) already noted in the diurnal profiles above.

### Forecasting model performance

All seven LSTM models were trained to stable convergence, with early stopping engaging between epochs 13 and 41 across fields. Training and validation loss curves confirmed no overfitting, with validation loss tracking training loss closely throughout, and no divergence observed at the point of early stopping (Fig. 10). Convergence speed varied by field: temperature stopped earliest, at epoch 13 (validation loss 0.0549), while rain required the most epochs, stopping at epoch 41 (validation loss 0.0162), consistent with rain’s noisier, more intermittent signal requiring more training to reach a stable minimum. The gas sensor fields (MQ-4, MQ-7, MQ-135) and soil moisture converged to the lowest absolute validation losses (0.0008–0.0033), reflecting their comparatively smoother, more autocorrelated series.

**Fig. 12:**
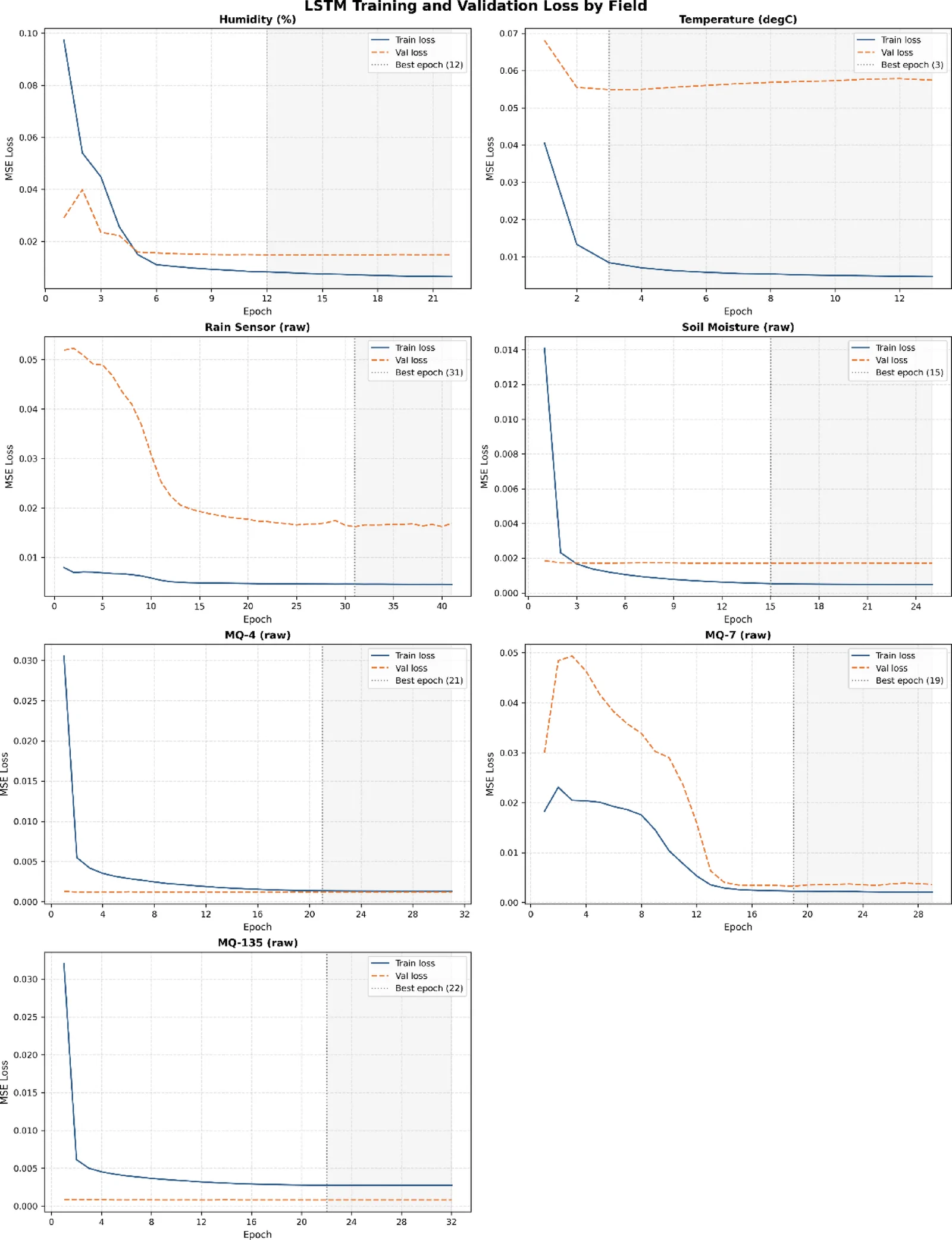
Training and validation loss curves for all seven univariate LSTM models.

Model performance on the held-out test set is summarized in Table 4. The gas sensor fields produced the strongest results (Table 5). MQ-4 achieved a MAPE of 0.81% (MAE 1.933 raw units), and MQ-135 achieved a MAPE of 0.31% (MAE 1.714 raw units), a narrow edge over a persistence baseline. Soil moisture achieved a MAPE of 6.51% (MAE 9.854 raw units); a persistence baseline outperformed the model on this channel (Table 6). MQ-7 carbon monoxide reached a MAPE of 5.66% (MAE 17.060 raw units), with a systematic underestimation of approximately 15 raw units throughout the test period, consistent with a minor distributional shift between the training and test portions of the series.

**Table 4:** Augmented dickey-fuller stationarity test results for all seven sensor fields.

Field	ADF statistic	p-value	Result	Modelling decision
Temperature	−3.9329	0.002	Stationary	Model in levels
Humidity	−4.1082	< 0.001	Stationary	Model in levels
Rain	−5.5272	< 0.001	Stationary	Model in levels
Soil moisture	−2.6472	0.084	Non-stationary	First-order differencing
MQ-4	−2.6226	0.088	Non-stationary	First-order differencing
MQ-7	−2.9575	0.039	Stationary	Model in levels
MQ-135	−2.1573	0.222	Non-stationary	First-order differencing

Temperature and humidity produced MAPE values of 11.11% and 10.20% respectively. Both models reproduced the correct diurnal frequency but underestimated amplitudes, attributable to the + 0.296 °C per day warming trend placing the test period partially outside the training distribution. The rain sensor MAPE of 46.42% is dominated by near-zero denominator values during dry periods; the MAE of 61.832 out of a 0 to 1023 ADC range is the more informative metric for this field, and predicted versus actual plots show that the model tracks the timing of raw rain-sensor fluctuations. 95% confidence intervals for each metric, computed via bootstrap resampling of the test windows (2,000 resamples), were narrow relative to the point estimates, with relative half-widths ranging from 0.17% to 6.64% across all fields and metrics. Predicted versus actual plots for all seven fields are shown in Fig. 11.

**Fig. 13:**
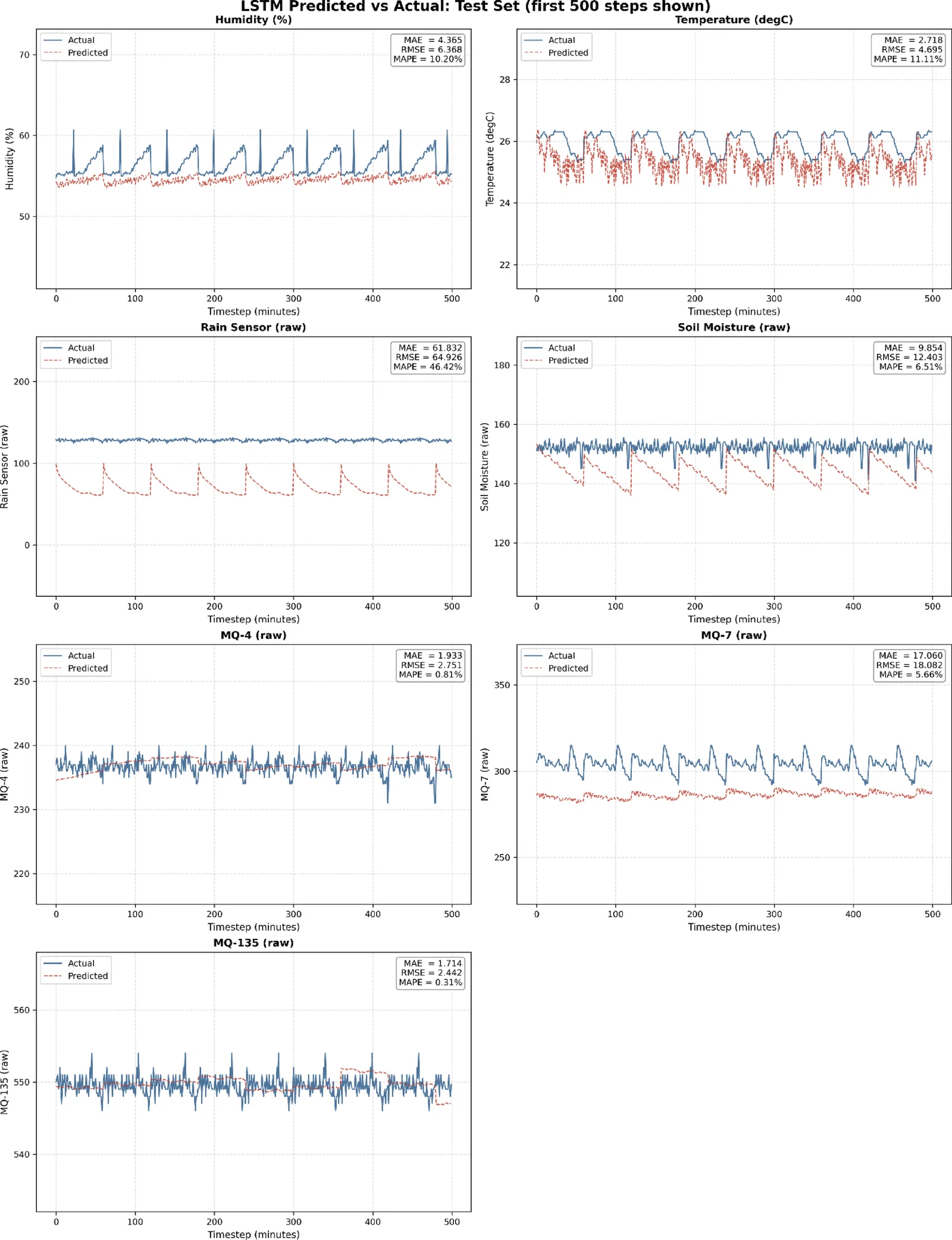
Predicted versus actual values on the held-out test set for all seven sensor fields.

### Comparison with naive forecasting baselines

To assess the incremental value of the LSTM forecasts relative to simple extrapolation, two naive baselines were evaluated on the same held-out test-set segments: persistence, which predicts the next hour equals the most recent observed value, and seasonal-naive, which uses the value collected 24 h earlier for its predictions. Table 6 presents the MAE, RMSE, and MAPE metrics for the baseline approaches alongside the LSTM findings from Table 5.

**Table 5:** LSTM forecasting performance on the held-out test set across all seven sensor fields.

Field	Sensor	MAE	RMSE	MAPE (%)	Notes
Temperature	DHT22 (°C)	2.718	4.695	11.11	Amplitude underestimation due to warming trend
Humidity	DHT22 (%)	4.365	6.368	10.20	Anti-phase underestimation consistent with temperature error
Rain	Resistive (ADC)	61.832	64.926	46.42	MAPE inflated by near-zero dry period values; MAE more informative
Soil moisture	Resistive (ADC)	9.854	12.403	6.51	Outperformed by persistence baseline
MQ-4	Methane (ADC)	1.933	2.751	0.81	Narrow edge over persistence baseline
MQ-7	CO (ADC)	17.060	18.082	5.66	Systematic offset of ~ 15 raw units noted
MQ-135	Raw (ADC)	1.714	2.443	0.31	Narrow edge over persistence baseline

**Table 6:** Comparison of LSTM forecasting performance with persistence and seasonal-naive baselines on the held-out test set across all seven sensor fields.

Field	LSTM			Persistence			Seasonal-naive		
	MAE	RMSE	MAPE (%)	MAE	RMSE	MAPE (%)	MAE	RMSE	MAPE (%)
Humidity	4.365	6.368	10.20	**3.132**	**5.912**	**6.67**	14.662	17.970	32.35
Temperature	2.718	4.695	11.11	**1.467**	**4.508**	**6.80**	5.833	8.153	23.05
Rain	61.832	64.926	46.42	**5.275**	**22.857**	**3.76**	10.656	39.755	7.91
Soil moisture	9.854	12.403	6.51	**3.561**	**8.459**	**2.52**	7.831	14.217	5.32
MQ-4	**1.933**	**2.751**	**0.81**	1.976	2.833	0.82	7.836	9.658	3.26
MQ-7	17.060	18.082	5.66	**3.895**	**6.444**	**1.29**	16.083	20.775	5.38
MQ-135	**1.714**	**2.443**	**0.31**	1.747	2.499	0.32	3.707	5.572	0.67

Persistence surpassed the LSTM according to the values of MAE, RMSE, and MAPE for humidity, temperature, rain, and MQ-7, with the gap for rain and MQ-7 particularly large, persistence attained around 12 and 4 times lower MAE, respectively. This trend aligns with the previously observed shift in the training distribution for these channels: the testing phase exhibited systematically elevated temperatures, reduced humidity, and higher rain-sensor and MQ-7 readings compared to the training phase, thereby skewing a static forecasting model while leaving a persistence forecast unaffected. Soil moisture exhibits a comparable trend in MAE. The MQ-4 and MQ-135 channels, exhibiting the most pronounced longitudinal patterns in Environmental Pattern and Trend Analysis, yielded the closest agreement between LSTM and persistence, with the LSTM slightly outperforming persistence across all three criteria. Seasonal-naive models exhibited inferior performance compared to persistence across all fields, suggesting that daily fluctuations over the 21-day deployment overshadow any recurring daily trends identified by a static 24-hour lag (Figs. 12, 13).

### Sustainable agriculture and environmental impact

The monitoring and forecasting pipeline presented here is intended as an early step toward the data-driven agricultural management envisioned under the UN 2030 Agenda for Sustainable Development^15^, particularly SDG 2 (Zero Hunger), SDG 6 (Clean Water and Sanitation), and SDG 13 (Climate Action). One-hour-ahead soil moisture forecasting (MAPE 6.51%) points toward anticipatory irrigation flagging based on a baseline learned from historical, site-specific data rather than a single fixed moisture value, since a static threshold is unlikely to generalize across different soil types, crops, and seasons. Validating forecasted moisture decline against real irrigation events and crop response, needed to establish this baseline, is identified as future work. MQ-135 forecasting (MAPE 0.31%) could similarly support flagging sustained increases in raw output for further attention, while the nocturnal MQ-4 peak at 23:00 may be relevant to future greenhouse gas monitoring applications. Together, these results demonstrate that a low-cost IoT platform integrating multi-sensor acquisition, cloud connectivity, and LSTM forecasting can provide a technically viable foundation for more precise and resource-efficient agricultural management, pending the agronomic validation identified as future work.

## Conclusion

This paper presented a low-cost IoT environmental monitoring and forecasting system deployed over 21 days at a residential garden site in the Nile Delta region of Egypt, demonstrating the technical feasibility of pairing affordable hardware with a cloud-connected data pipeline and deep learning forecasting. By combining an Arduino Uno R3 and ESP8266 NodeMCU Wi-Fi module with seven sensor channels spanning atmospheric, soil, and gas-phase measurements, the system achieved real-time data acquisition at an average interval of approximately one minute, with a preprocessing pipeline retaining 98.48% of the exported record. The deployment paired this physical monitoring infrastructure with seven univariate LSTM models trained on ADF-conditioned time series, producing one-hour-ahead forecasts across all sensor channels simultaneously. Benchmarked against persistence and seasonal-naive baselines, however, the LSTM held a modest edge over persistence only for methane and air quality forecasting; persistence matched or outperformed the LSTM for the remaining five channels, a result consistent with a verified train/test distribution shift in the atmospheric and gas channels.

Future work will prioritize extending the deployment to cover a full seasonal cycle, directly addressing the training distribution limitation identified for atmospheric channels, and validating the soil moisture forecasting approach against real irrigation events and crop response to establish an adaptive, historically informed moisture baseline in place of a fixed threshold. Incorporating multivariate LSTM formulations to exploit the cross-sensor relationships documented in the trend and diurnal analyses and migrating inference to an edge device to reduce cloud dependency, represent the most impactful next steps toward a fully autonomous, low-latency precision agriculture system.

## Data Availability

The dataset generated and analyzed during this study is available from the corresponding author upon reasonable request.

## Declarations

### Software

The data acquisition firmware was developed using the Arduino IDE. Data logging and cloud visualization were performed using the ThingSpeak IoT platform. Statistical analysis and LSTM modeling were implemented in Python 3 using the pandas, NumPy, statsmodels, SciPy, and PyTorch libraries.
